# Shear-enhanced dynamic permeability development of magma vesiculating in cylindrical conduits

**DOI:** 10.1038/s41598-026-43344-8

**Published:** 2026-03-24

**Authors:** J. Birnbaum, J. Schauroth, J. Weaver, J. E. Kendrick, A. Lamur, Y. Lavallée

**Affiliations:** 1 Department of Earth and Environmental Sciences, Ludwig-Maximilians-Universität München, Theresienstraße 41, 80333 Munich, Germany; 2 Earth, Ocean and Ecological Sciences, University of Liverpool, 4 Brownlow Street, Liverpool, L69 3GP UK

**Keywords:** Magma vesiculation, Bubble shear, Porosity, Permeability, Engineering, Materials science, Solid Earth sciences

## Abstract

During magma vesiculation, permeability is established when growing bubbles form connected networks which allow fluids to percolate. This percolation threshold controls the relative rates between magma ascent and volatile outgassing, which in turn dictate eruptive style. Percolation in crystal-poor magmas is controlled primarily by vesicularity and shear. We investigate the effect of realistic shear conditions on system-spanning connectivity via vesiculation experiments on rhyolitic melts in a cylindrical, conduit-like geometry. The amount of shear experienced is controlled by varying the sample and confining diameters to allow for various degrees of free (isotropic) followed by confined (anisotropic) expansion. We observe two regimes of behavior for samples dominated by (1) initial isotropic vesiculation, in which the onset of permeability corresponds with the beginning of shear deformation that connects existing vesicles and (2) anisotropic vesiculation which shows low percolation thresholds (< 20%) followed by continuous deformation. We find distinct trends in porosity and permeability for sheared and unsheared samples. Furthermore, We recover the bulk dynamic permeability time series in the shearing melts which requires maintaining connectivity both within bubbles and to the exterior. The development of anisotropy is ubiquitous in vesiculating magmatic networks and our data highlight the importance of considering the in situ shear conditions when investigating the percolation threshold.

## Introduction

The transport and eruption of magma is driven by buoyancy, imparted primarily by volatile exsolution. Examining the eruptive record, eruptions of all sizes can shift between effusive and explosive modes, or exhibit a combination of the two (e.g.,^1,2^), depending on the ability and efficiency of magma to release gas pressure^3–7^, controlled by its vesicularity and, importantly, permeability. It is therefore crucial to understand the processes and timescales of volatile loss from magma via vesiculation (the combination of bubble nucleation, growth, and coalescence), percolation, and outgassing.

The solubility of volatiles (e.g., H$_2$O, CO$_2$) in magmas is dependent on magma chemistry, pressure, and temperature. During eruptions, magmas rapidly decompress, reducing the solubility of volatile species, prompting exsolution^8^. Shear-induced heating may also be a mechanism for reducing the solubility of volatiles during eruptions^9^. Detailed analytical and numerical models simulate the process of bubble growth (e.g.,^10–15^). As bubbles grow, the melt films separating them may thin and eventually rupture, resulting in bubble coalescence. Although coalescence is a process of considerable interest, the physical processes of bubble coalescence are complex (e.g.,^16^), and both crystallinity^17^ and shear conditions^18^ likely play important roles.

As bubbles grow closer and interact or coalesce, it becomes possible for gas to flow between bubbles (e.g.,^19,20^). This percolation allows for permeable outgassing and subsequent densification of the magma in the “permeable foam” model of silicic magma transport^4,21–23^. Idealized magmas are predicted to have a critical porosity of $\sim$30–64% to allow percolation^24,25^. Laboratory vesiculation experiments have found a higher threshold for connectivity, attributed to the non-random locations of bubbles during nucleation and growth, or viscous barriers to coalescence^26,27^. Furthermore, uncoalesced bubbles in close proximity may take on polygonal shapes, which can support very high volume fractions of gas without inducing connectivity, which are observed in some volcanic eruption products (e.g., reticulite).

In reality magmas generally contain variable fractions of bubbles and crystals with complex shape and size distributions, and their properties are scale-dependent and rarely homogeneous or isotropic. Measurements of natural and experimental samples show percolation thresholds between $\sim$30–80% (e.g.,^28^). The percolation threshold and permeability of volcanic rocks vary as a result of shear deformation^2,18,29–31,33^, strain localization^34^ and fracture development^35–41^, melt chemistry^42^, magma rheology^16,43^, the presence of crystals^44,45^, the distribution of bubble and crystal microstructure^28,43,46,47,48^, and time^49^.

Beyond the percolation threshold, permeability shares non-linear relationships with porosity (which may consist of vesicles, intergranular voids, and cracks). In natural materials, large variations in permeability exist for a given porosity, owing to the complex eruptive processes leading to the construction of porous networks^19,46,50,51,52,53^. Effusive and explosive eruptive products exhibit broadly distinct permeability-porosity relationships, underlined by differing dominant porosity-controlling mechanisms with a hysteresis effect^20,31,42^; explosive products display permeable pathways apparently controlled by bubble growth and coalescence, which can be modeled by percolation theory, whereas effusive products have permeable pathways controlled by shear, bubble elongation, compaction, fracturing, and welding, and are better approximated by a combination of capillary tube ( e.g., via the Kozeny-Carman relationship;^48,54^) and fracture models^19^.

In natural systems, the processes of porosity creation and destruction will vary in space and time, with profound impacts on eruption rate and style. This will result in variable vesicle textures, reflecting, among other processes, the distribution of shear imparted on vesicular magmas during emplacement, even within a single eruptive unit (e.g.,^29^) which capture only the final state of the cooled lava. Understanding the complex evolution of porosity and permeability in response to vesiculation, shear, and outgassing remains an outstanding challenge. Despite its relevance to volcanological settings, the development of permeability in conduit-like geometries remains understudied. Vesiculation-driven deformation in conduits results in a combination of pure shear in the direction of elongation, combined with simple shear due to conduit friction, whose rate of deformation is driven by buoyancy and the bubble overpressure which decrease in response to the establishment of permeable pathways. This differs from, for example, rotational shear experiments (e.g.,^18,32^) which are dominated by simple-shear, and have a different distribution of shear rate across the sample from interior to margin and top to bottom; or from the addition of uni-axial compression, which gives a complicated shear rate distribution with a contribution of pure shear driving bubble collapse^31^, which result in bubbles oriented largely circumferentially and sub-perpendicular to the direction of interest.

**Fig. 1:**
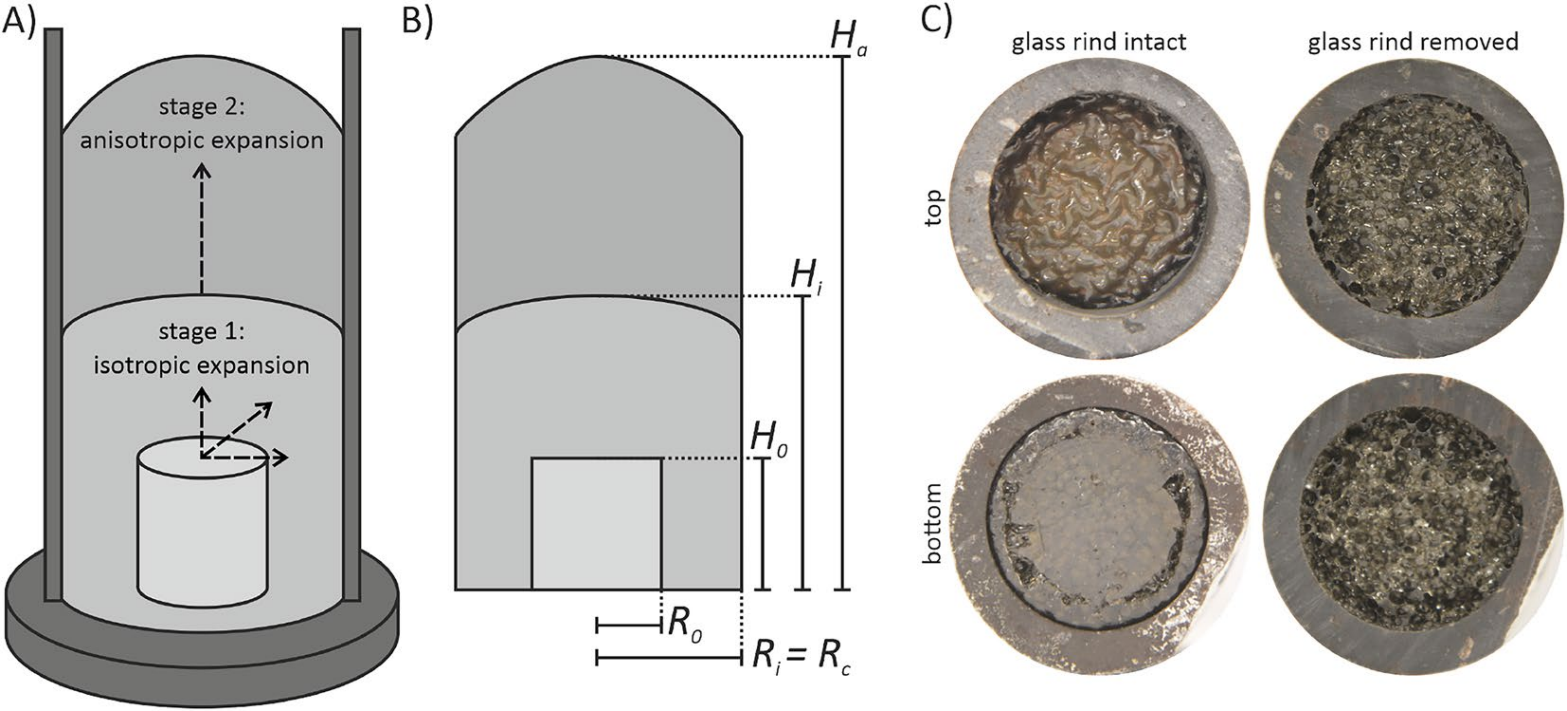
A) Sketch of experimental setup for confined experiments; B) definitions of radius and height for the initial sample ($R_0$ and $H_0$, respectively), the maximum extent of isotropic expansion ($R_i$ and $H_i$), and the anisotropic expansion ($R_a$ and $H_a$); C) photographs of post-experimental samples from above and below, before and after removing the glass rind.

We studied the development of permeability in vesiculating cylindrical samples, either unconfined, or placed inside cylindrical crucibles of variably larger diameter (Fig. 1). Our experiments allow for simultaneous vesiculation, permeable flow, and shear, directly analogous to the shear conditions present in volcanic conduits for centimeter-scale samples. Our study substantially expands both the number (106) and absolute size (1-2 cm, about 20 times the typical bubble diameter) of vesiculation experiments in cylindrical geometries^30,33^. Additionally, by varying the proportion of initially isotropic expansion, followed by anisotropic expansion via the ratio of sample to conduit diameter (C), we control total amount of shear, and the vesicularity at shear onset, that result in different bubble shape distributions and porosity and permeability evolution. Finally, we discriminate between the porosity of the cooled, post-experimental, samples from the bulk outgassing behavior of the molten suspension and underscore the importance of system-spanning, rather than local, connectivity and permeability.

## Results

### Bubble textures

**Fig. 2:**
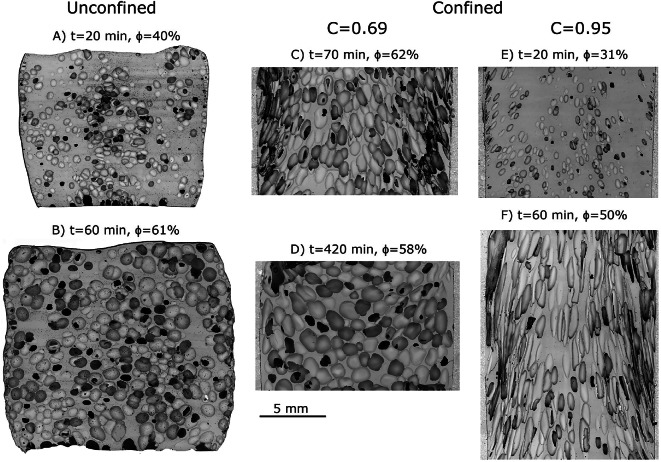
Backscatter electron images of unconfined samples (**A**, **B**) and confined samples (**C–F**). Unconfined samples (**A**) during early vesiculation and (**B**) with increasing dwell time and total porosity. For a confinement ratio of C = 0.69 at (**C**) 70 min at the beginning of shear, and (**D**) 420 min after significant shape relaxation and minor porosity reduction. For a higher confinement ratio of C = 0.95 at (**E**) 20 min capturing shear during early vesiculation, and (**F**) 60 min showing volume loss due to degassing during shear at higher porosity. Note the similar dwell times for **B**, **C**, and **F** and their contrasting textures due to confinement.

Bubble networks in confined and unconfined experiments exhibit textural differences (Fig. 2). In the unconfined samples we see nearly equant bubble shapes with increasing bubble size at increasing dwell time, resulting in higher porosity. In direct contrast, a partially confined sample (C = 0.69) after nearly the same dwell time results in a similar total porosity, but shows highly distorted bubbles aligned sub-parallel to the flow direction at the margins and with slightly elongate and larger bubbles in the center aligned with the flow direction (Fig. 2B,C). At higher degrees of confinement (C=0.95), the sample exhibits bubbles with higher aspect ratio, closer alignment to the flow direction, and modestly lower total porosity (Fig. 2F). Early contact with the crucible results in bubble elongation in even very small bubbles early in the vesiculation process (Fig. 2E). In contrast, for the sample that experienced a much longer dwell time (420 min, Fig. 2D), shear has stopped and the bubbles have relaxed to near-round shapes. The bubble orientations suggest that the velocity in the center of the sample may have reversed direction with either gas loss, or simply through shape relaxation of the interior bubbles.

We measure the number density manually in each of the images and perform stereological corrections after^55^. The samples of short duration ($\le$ 70 min) are all consistent with one another at $\sim2-4\times10^{10}$ 1/m$^3$, normalized to the melt volume, independent of confinement ratio. Only the long-duration sample (420 min) shows a substantially reduced bubble number density of $\sim7\times10^{9}$ 1/m$^3$, indicative of coalescence.

### Porosity of unconfined vesiculation experiments

**Fig. 3:**
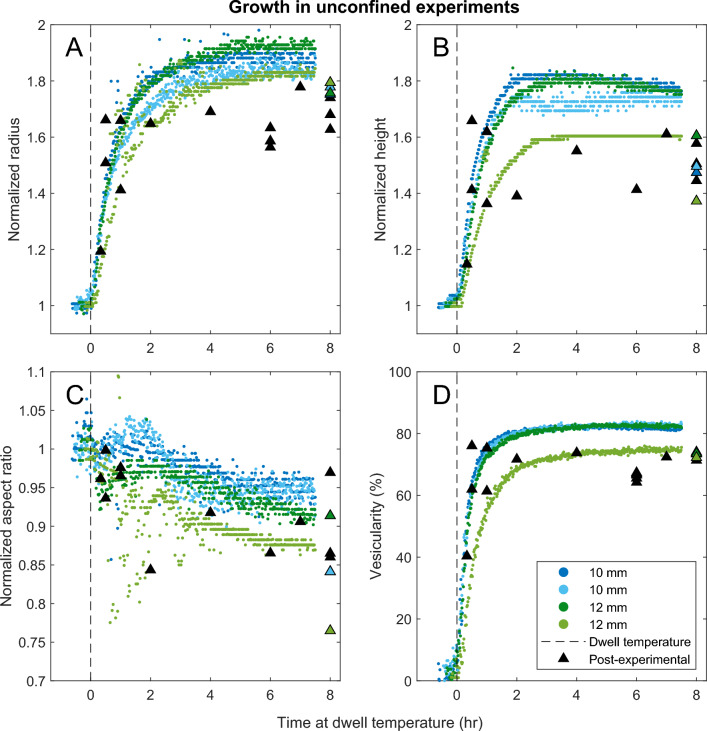
Sample dimensions measured from thermal infrared imaging of unconfined experiments showing changes in (**A**) normalized radius, (**B**) normalized height, (**C**) normalized aspect ratio, and (**D**) vesicularity. Small markers show each frame colored by experiment number. Blue samples had an initial radius of 10 mm and green samples had an initial radius of 12 mm, and all were unconfined. Triangles show post-experimental analyses colored for the same experiments and black for experiments not imaged. Measurement uncertainty for the post-experimental samples is smaller than the marker size. The dashed line in all panels indicates the time at which experiments reach the dwell temperature of 1009 $^\circ$C.

We vesiculated samples of aphyric rhyolitic obsidian in unconfined geometries to develop a baseline for how unsheared samples behave. We observe that vesiculation begins at a temperature of $\approx$950 $^\circ$C, 10 min before we reach the dwell temperature of 1009 $^\circ$C. By the time we reach the dwell temperature, the samples already contain 5-10% vesicularity. At the dwell temperature, the vesiculation rate accelerates and then decreases, with most of the vesiculation occurring within 100 min. Samples reach a maximum vesicularity of 80-85 vol%. The volumetric increase is accommodated by a near doubling of both radius and height (Fig. 3). With increasing time, the samples continue to expand laterally at the expense of vertical growth, showing a gradual decrease in the aspect ratio.

### Porosity and permeability of confined experiments

**Fig. 4:**
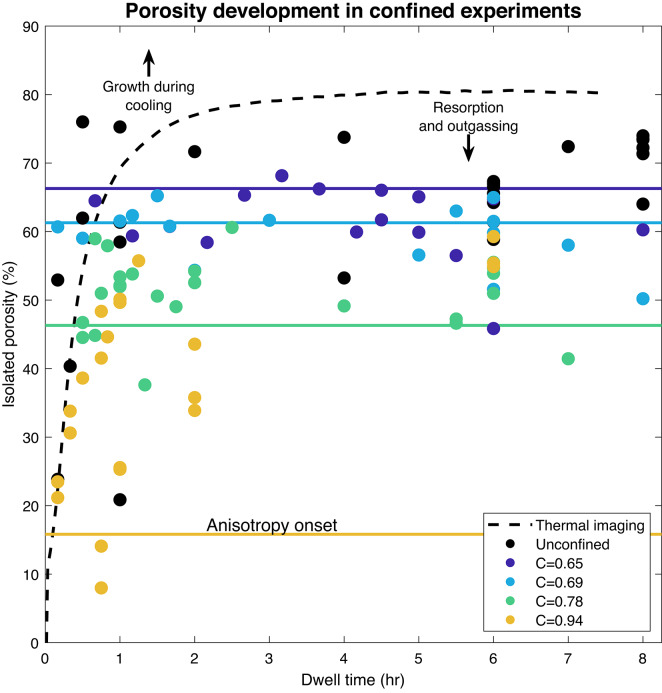
Porosity measurements of post-experimental samples with variable dwell times and different degrees of confinement indicated by different colors. Measurement uncertainty for the post-experimental samples is smaller than the marker size. The average porosity measured from thermal infrared imaging for unconfined samples (black) is shown in the dashed line. From the processed imaging results, we calculate the porosity required for the sample to reach the crucible resulting in anisotropy onset, shown in colored lines. Deviation of the post-experimental samples from the imaging indicate additional degassing or bubble volume changes and thermal contraction during the early part of cooling.

**Fig. 5:**
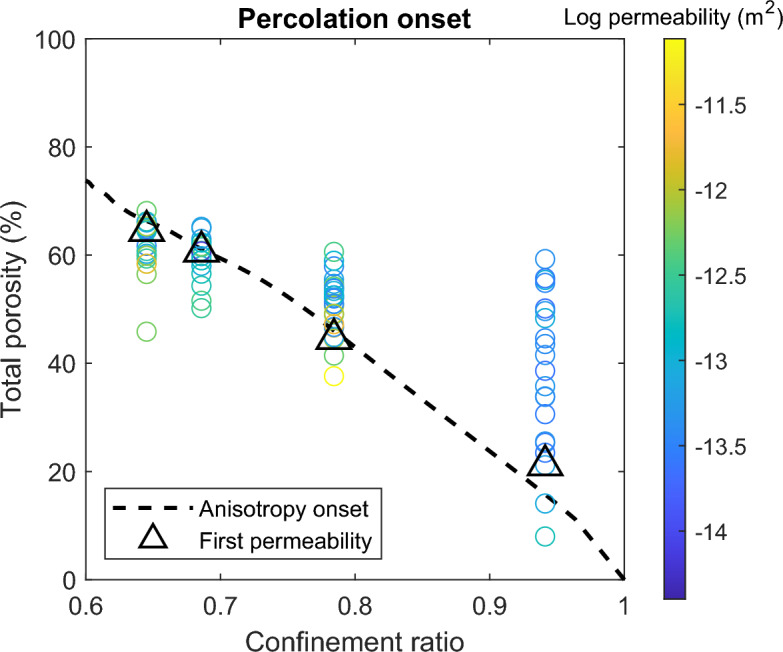
Porosity resulting in significant permeability for different confinement ratios, colored by permeability. The black triangles highlight the experiments with the shortest dwell time that produced significant permeability ($>10^{-15}$ m$^2$). We interpret these samples to represent the onset of percolation. Samples at longer durations maintain permeability at lower porosity than the initial onset (Fig. 6), which are visible below the black triangles in this figure. The dashed black line indicates the porosity required for the sample to first reach the crucible, but all samples shown here are coupled to the crucible.

**Fig. 6:**
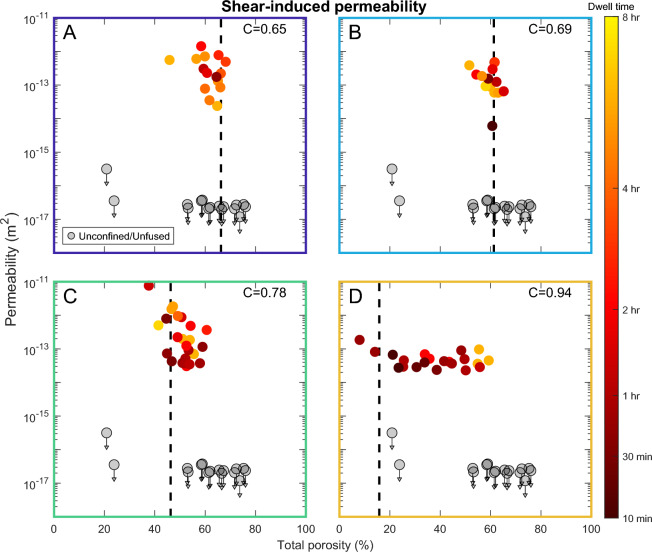
Total porosity and permeability measurements for different degrees of confinement: (**A**) C = 0.65, (**B**) C = 0.69, (**C**) C = 0.78, and (**D**) C = 0.94. Measurement uncertainty for the post-experimental samples is smaller than the marker size. Dashed lines indicate the calculated porosity at contact with the crucible. Measurements are colored by dwell time. Grey samples were unconfined or did not grow sufficiently to contact the crucible walls. Arrows indicate that measured values are maxima for permeability measurements terminated prior to equilibration (unfused).

**Fig. 7:**
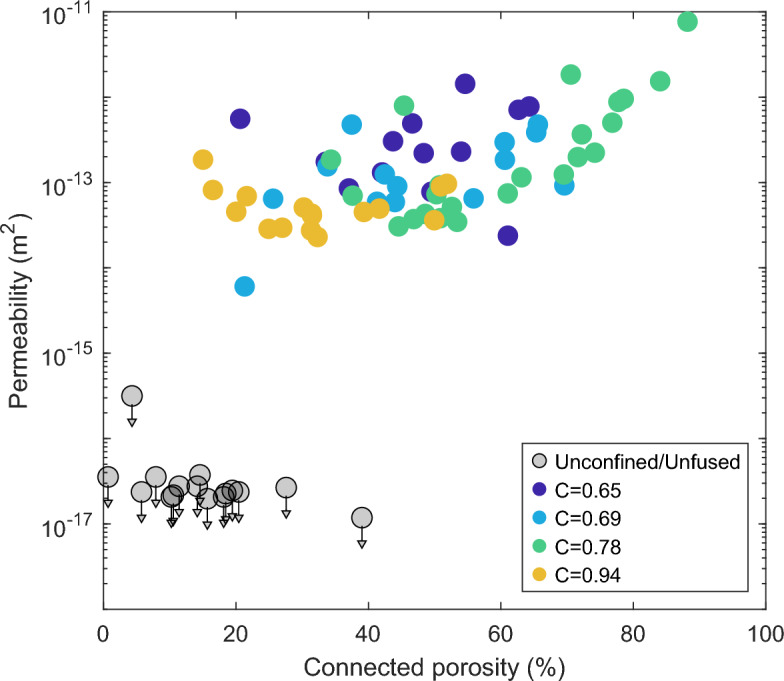
Permeability as a function of connected porosity and confinement ratio (color). Arrows indicate that measured values are maxima for permeability measurements terminated prior to equilibration. We observe a distinct separation between sheared and unsheared (unconfined/unfused) samples.

The final total porosity of the confined samples is consistently lower than for unconfined samples, with larger differences for samples with more confinement (Fig. 4). We expect the creation of porosity during initial vesiculation to be similar between the confined and unconfined samples, suggesting sheared samples show reduced porosity as a result of outgassing due to permeable flow. From the sample heights and porosities, we estimate the total final shear strain and average strain rates which are up to 270% and 8$\times 10^{-4}$ 1/s. We observe two different regimes depending on the confinement ratio, $C=\frac{r_{\text {sample}}}{r_{\text {crucible}}}$. In cases of low confinement (C = 0.65 and 0.69), final total porosities rarely exceed the porosity required for the onset of anisotropy, i.e. touching the edges and shearing upward (61 and 66%, respectively) and the majority of vesiculation occurs during the isotropic stage of growth. When the samples contact the crucible, porosity remains nearly constant afterward, while unconfined samples continued to grow after the same dwell times (up to $\sim$72%). At higher confinement (C$\ge$0.78), the samples reach the crucible earlier and continue to increase in porosity after the onset of anisotropy. The onset of permeability is at lower porosity with more confinement (46% for C=0.78 vs. 61% for C= 0.69; Fig. 5), but the permeability during onset is similar to the more porous, lower confinement samples. The samples at C=0.94 show a clear progression with increasing dwell time leading to higher porosity, but permeability either decreases or remains stable with further degassing (Fig. 6). In all cases, the lowest porosity sample which shows connectivity occurs after a longer dwell time and exhibits higher permeability than that at which permeability is first established. We observe either no, or a mild inverse, correlation between porosity and permeability. This relationship is subtle, because porosity remains nearly constant between samples of the same confinement, especially in the isotropic-dominated regime. If instead we consider the role of connected rather than bulk porosity, we find a positive overall trend with permeability, with little separation due to the degree of confinement other than the overall lower porosity present in the C=0.94 samples (Fig. 7).

## Discussion

### Permeability hysteresis

To a first order, permeability is expected to increase with increasing porosity, with shear lowering the threshold for connectivity. We clearly observe the second prediction. So why, then, do we observe an apparent decrease in permeability with increasing total porosity at a given confinement ratio (e. g., Fig. 6D)? The Kozeny-Carman model idealizes permeable flow through porous media as flow confined to pore channels that transverse the sample through tortuous paths that offer resistance proportional to the friction along the channel walls, offering a direct link between pore geometry and permeability. Many approximations exist for various geometries, for example:

1 $$\begin{aligned} k = K\frac{\phi ^{m}}{(1-\phi )} \, \end{aligned}$$

from^54^. In practice, calculations for *K* and *m* only exist for highly simplified geometries and are fit empirically to data. We expect these to vary with the connected porosity, characteristic bubble and pore aperture sizes (conductance), tortuosity, and specific surface area (itself a function of the preceding). Tortuosity is typically defined according to the ratio of the path length the percolating fluid must travel compared to the sample length, where increasing tortuosity acts as an additional impediment to flow and decreases permeability. Our permeability measurements roughly overlap with the “effusive” region identified in ^19^, and with existing compilations^46,56^.

The compilation of connectivity data in ^46^ shows that in natural samples, especially from lava flows which experience more shear, connectivity typically rapidly increases over a narrow porosity interval and then remains nearly constant between about 0.8-1. That is, above the threshold for connectivity, pores remain nearly all connected with a hysteresis in which connectivity persists during bubble relaxation or collapse, even to low porosities. Indeed, our sheared samples show nearly complete connectivity, while the unsheared samples uniformly have low connectivity ($\lesssim$0.5). When we compare connected porosity with permeability, we do observe a broad positive correlation across all degrees of confinement in sheared samples (Fig. 7), with a clear separation from the unsheared samples. However, within the C=0.94 samples, the pores are nearly all connected, suggesting that the mild inverse correlation between total porosity and permeability is controlled by a factor other than connectivity (Fig. 6, Supplementary Table S2).

We may expect that tortuosity, in the direction of the fabric, should decrease with the increasing anisotropy of the sample^53^. While we discuss permeability as a single value for each sample in this paper, in reality permeability is a tensor and depends on the direction of flow. Because all our samples are measured in the same orientation, with flow through the cylindrical crucible, the direction of permeability measurement is parallel or sub-parallel to the direction of bubble elongation. We might therefore expect that with increasing anisotropy (shear), samples should display higher permeability even at the same porosity and connectivity. This would suggest that samples with the same porosity but greater confinement should show higher permeability. This trend is not evident in the data. Furthermore, given the continued high permeability at the longest times which have experienced significant bubble shape relaxation (Fig. 2D), tortuosity does not seem to play a major role in explaining the variability in permeability of these samples, perhaps because the pore connectivity structure partially remains during relaxation without dramatically increasing tortuosity. Other studies have found a low correlation between tortuosity and permeability (e.g.,^57^).

Finally, we turn to pore apertures: the cross-sectional area of the channels through which fluid flows. In bubbly suspensions, this usually relates to the aperture size between connected bubbles, which increases with increasing bubble size and coalescence^48^. We do observe a continued increase in bubble size with time in our experiments (Fig. 2). Highly elongate bubbles will potentially have smaller pore apertures compared to a rounded bubble of the same volume/equivalent diameter due to the smaller radius of curvature at their tips^58^. This provides a mechanism for maintaining high permeability in the low confinement ratio experiments and explains some of the hysteresis in which shear promotes the initiation of widespread connectivity, which is maintained during bubble shape relaxation at longer dwell times. This is also supported by the higher permeability observed at the onset of percolation in the low confinement samples which must already have large bubbles at the time of shear-induced connectivity.

Changes in the pore geometry would result in different apparent fitting factors in the Kozeny-Carman relationship for each sample with its own unique history. We would not expect one curve to explain the variability in our data, but rather a family of curves specific to the heating and shear history experienced by different samples. For example, surface area for a pore increases with increasing elongation, which at the same tortuosity would approximately halve permeability for bubbles with an aspect ratio of 10 compared to spheres. Furthermore, permeability through spherical pores should scale with the aperture radius to the fourth power^48^, meaning our measured variability in the permeability of $\sim$1–2 orders of magnitude at a given porosity could be explained by a factor of only $\sim$2 increase in aperture radius. Further investigation of the relationship between pore elongation and pore throat size and resultant permeability appears a fruitful direction for future work in volcanic materials.

### Regimes of bubble growth and outgassing

Like many other studies on permeability development in magmas and lavas, we are limited to measurements of permeability in cooled and solidified rocks which necessarily do not have the same dynamics as a melt. Each sample represents a snapshot of a particular time and pore geometry. Fortunately, we can combine our permeability measurements with the development of porosity to understand gas transport at high temperatures. If the melt is relaxed (as suggested by the observed changes in bubble shape and lack of fractures in the BSE images), then the volume change of the pore space reflects the mass balance of water vapor in the bubbles. Diffusion of water from the melt is the only source of water mass for bubble growth in these experiments, and should be relatively similar across different degrees of confinement, with perhaps slightly faster diffusion into elongate bubbles which have a higher surface area and into bubbles with lower overpressure.

We can consider the relative contribution of different water loss mechanisms. The experiments of ^59^ demonstrate the importance of the thin dehydrated rind in retaining vapor bubbles in the melt phase. Water diffusion through this rind can reduce the porosity of the sample with sufficient time (hours to tens of hours), and particularly for small samples ($\lesssim$3 mm diameter)^60^. If dehydration through this rind was the dominant water loss mechanism, we would expect that the samples with the greatest surface area exposed to the air (unconfined samples) would show the greatest gas loss, this volume loss would be apparent in Fig. 3, and to be volumetrically-important, the dehydrated rind would be of resolvable thickness (Fig. 2). Since this is not the case, the gas must be lost via permeable pathways connected to the exterior of the sample. Perhaps shear along the margins disrupts the rind formation and allows for some permeable outgassing.

**Fig. 8:**
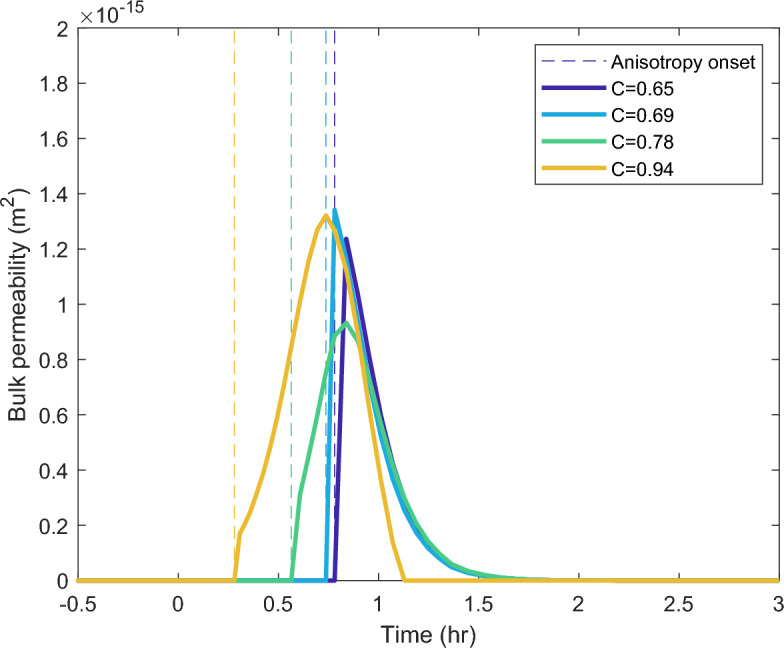
Calculation of dynamic bulk permeability recovered from time series experiments and numerical simulation. Color indicates the confinement ratios, while the dashed lines indicate the onset of anisotropy. Time is measured from t=0 when the experiment reaches the dwell temperature.

We employ the vesiculation model described above^10^ to calculate the mass of water into the bubbles through time, the viscous resistance provided by the melt, $\langle \mu \rangle$, and to correct for the vesicle shrinkage upon cooling. We fit a curve, informed by the shape of the modeled curve, to pass through the data from the post-experimental samples at each confinement ratio to recover the vesicularity, and consequently bubble size during time in the sheared experiments. Because the rate of bubble growth is proportional to the overpressure in the bubble via:

2 $$\begin{aligned} \frac{\partial R}{\partial t} = \frac{1}{12 R^2 \langle \mu \rangle }\left(P_b - P_{\text {atm}} - \frac{2\Gamma }{R}\right) \, , \end{aligned}$$

from the bubble radius through time, we can compute the pressure in the bubble, which relates to the mass through the water vapor equation of state. From this mass loss and the pressure difference between the bubble and atmospheric pressure, we can calculate the bulk Darcy permeability through the sample. Figure 8 shows the dynamic bulk permeability for each confinement ratio which further highlight the difference between our two regimes.

**Fig. 9:**
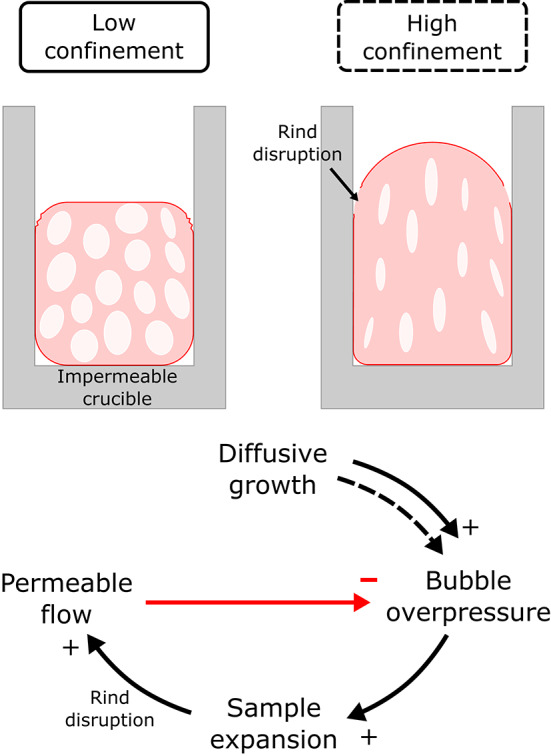
Feedbacks between bubble growth due to water diffusion and outgassing through permeable flow along the disrupted rind. Black arrows represent reinforcing relationships and red arrows represent mitigating relationships, the dashed arrow highlights the extra role of diffusive growth in high confinement geometries.

In the low confinement experiments (C$\le$0.78) permeability and connectivity developed rapidly after the onset of shear, and this permeability development during the end stages of vesiculation seems to be sufficient to relieve bubble overpressure and halt further growth (Fig. 8). If rind disruption is a crucial part of connecting the sample interior to the surrounding atmosphere, we might expect permeable gas flow to end when the sample expansion stops and the rind is able to re-form and persist. For these samples, system-scale permeability is likely a transient behavior at high temperatures with a feedback between bubble growth and permeable gas loss which are roughly balanced (Fig. 9) resulting in a dynamic equilibrium porosity or potentially episodic connectivity and outgassing. As the samples continue to mature with bubble coalescence and shape relaxation, which change the permeability of the cooled and cut samples, they are likely isolated from the atmosphere by the rind.

When the onset of shear occurs early in the vesiculation (C$\ge$0.78) bubble growth continues after shear, albeit at a suppressed rate, in which water diffusion into the bubble is still faster than water loss. In the case of C=0.78, we observe an intermediate behavior with a rapid increase in permeability at the onset, followed by a more gradual increase in bulk dynamic permeability several minutes after the onset of percolation. The continued flow of the material potentially reduces the sealing effect of the rind on the sample’s free surface. In the highest confinement ratio samples (C $\ge$ 0.94), we observe a gradual increase of permeability accompanying a slower vesiculation path which reaches lower final values of porosity (Fig. 4) because a partially open system is maintained for a longer portion of the vesiculation history. In this regime, despite the development of system-spanning connectivity, vesicularity increases at a suppressed rate, which cautions against assuming nearly-instantaneous vesicle loss and densification upon connectivity in natural systems of high viscosity. Instead, the competition in mass balance between diffusive volatile delivery in, and degassing out due to connectivity, combined with a pressure gradient to drive flow should be considered, all of which must be compared to the ability of the melt surrounding the bubbles to relax.

The maximum calculated dynamic bulk permeability is approximately two orders of magnitude lower than the permeability of the cut post-experimental samples. Some of this deviation may reflect changes during continued textural evolution and cooling. However, the 1-3 orders of magnitude difference between these quantities supports the idea that only a localized connectivity path exists through the torn rind at the margins, compared to the entire surface area exposed in the cut samples. The difference in permeability is consistent with syn-experimental outgassing though only the outer $\sim$5% of the radius. The dynamic permeability trends to zero once the rind is able to form across the whole sample cross section.

In our experimental setup, the choice of basalt crucibles may play an important role in retaining the bubbles except through the torn rind at the sample surface. The basalt itself has a very low permeability which limits outgassing along the bottom and sides of the sample. Porous ceramic crucibles have been demonstrated to allow such gas escape^61^. This highlights the importance of understanding the rock properties of conduit walls in natural systems which may play an important mediating role in allowing gas escape during magma ascent, which has been highlighted by numerous conduit ascent models (e.g.,^6,7^).

### Implications for eruptive behavior

Our experiments produce vesicular networks sheared at average shear rates up to 8$\times 10^{-4}$ 1/s, with instantaneous rates potentially an order of magnitude higher. This falls within the large range of shear rates expected for effusive and low-explosivity (e.g., vulcanian) eruptions, $\sim10^{-7}-10^{0}$ 1/s, considering conduits of $\sim10^{1}-10^{2}$ m (e.g.,^62–65^). The shear-rate distribution across cylindrical conduits scales directly with the radius for homogeneous (but, not necessarily Newtonian) fluids. This suggests that our findings for the percolation of fluids are directly relevant to local (at least cm scale) flow in volcanic conduits. We find that small total strains ($\sim10^{-2}$) in the high capillary regime are sufficient for establishing connectivity and allowing percolation in vesicular networks with more than $\approx$0.4 porosity, with a hysteresis effect in which connectivity remains even after shear is removed and bubbles are allowed to partially relax in shape. However, connectivity between bubbles is not, alone, sufficient to produce rapid outgassing which requires system-spanning connectivity and a pressure gradient between the bubbles and the surroundings. Outgassing through wall rocks (e.g.,^4,6,7^) has long thought to be a control on eruption rate and style, in which hydraulic fracturing may be an important mechanism for creating permeable pathways away from the magma^66,67^. Together our results emphasize that while percolation onset may occur early during magma ascent, outgassing behavior may be controlled by external factors and the dynamic feedback between bubble pressure and texture.

## Conclusions

Our experiments document the importance of shear on establishing bubble connectivity and permeability in silicate melts. We find that during vesiculation in the absence of shear, the percolation threshold is very high, in fact all of our samples, even up to our maximum porosity of 76%, show permeability less than $10^{-15}$. After only small amounts of shear, connectivity is established quickly. In samples that reached 60-70% vesicularity, permeability is established upon the onset of shear and post-experimental samples exhibit overall high values of permeability between 2$\times$10$^{-14}$ and 2$\times$10$^{-12}$. In samples with lower porosity and more rapid shear, permeability is established at lower porosity (<20%), but with a lower permeability 2$\times$10$^{-14}$-2$\times$10$^{-13}$. Under shear, bubble connectivity is established and remains present even after the end of shear and significant bubble shape relaxation following the end of vesiculation ($\sim$2 h). Instead, permeability in sheared samples is likely a function of both connected porosity and conductance (specific surface area, tortuosity, aperture radius) with changing bubble size and aspect ratio. Reconstruction of the outgassing history demonstrates that bulk dynamic permeability feeds back into bubble growth and is sensitive not only to internal pore space connectivity, but also to the connection to the atmosphere which may have significant edge effects.

## Materials and methods

### Experimental setup

We studied the development of permeability in vesiculating magma using two test methods (1) unconfined vesiculation promoting isotropic expansion, and (2) variably confined vesiculation promoting momentary isotropic expansion followed by different extents of anisotropic expansion. For both test types we prepared and used cylindrical cores of aphyric rhyolitic obsidian from Hrafntinnuhryggur ridge at Krafla volcano (Iceland), with diameters and heights (1:1 ratio) of 10 mm and 12 mm. For confined experiments, samples were enclosed in cylindrical shells (i.e., hollow tubes) of dense, holocrystalline basalt from Seljavellir (Iceland), as the material is stable at the experimental conditions and has a low permeability.

### Characterisation of obsidian samples

Whole-rock geochemistry of the obsidian was determined by X-ray fluorescence (XRF) in a PANalytical Axios Advanced XRF spectrometer at the University of Leicester. Major elements were measured on glass beads fused from ignited powders using a sample to flux ratio of 1:5 (80% Li metaborate: 20% Li tetraborate). Results are reported as component oxide weight percent and have been recalculated to include loss on ignition (LOI).

The water concentration dissolved in the rhyolitic glass was measured by Fourier-transform infrared spectroscopy (FTIR) using a Thermo Nicolet 380 FT-IR and Nicolet Centaurus microscope at the University of Liverpool. Doubly-polished wafers were prepared with a thickness of $\sim$130 $\mu$m, determined with a high- precision caliper. The spectra of four spots, $\sim$100 $\times$ 100 $\mu$m, were acquired and analyzed. H$_2$O$_t$ concentrations were calculated by measuring the absorbance of the $\sim$3500 cm$^{-1}$ peak above a linear background and using the Beer-Lambert law, with a molar absorptivity coefficient of 90 mol$^{-1}$ cm$^{-1}$^68^ and a dry powder density of 2400 kg m$^{-3}$, measured by helium pycnometry.

The glass transition temperature, T$_g$, was determined by differential scanning calorimetry (DSC) in a Netzsch simultaneous thermal analyser (STA) Jupiter 449 F1 at the University of Liverpool, using a 1 mm thickness, 6 mm diameter disk of rhyolite weighing 67 mg. Each test was initially performed on an empty lidded platinum crucible to provide a correction baseline, followed by measurement on the sample. The assembly was heated to 1000 $^\circ$C at 10 $^\circ$C min$^{-1}$ in the presence of a 20 mL min$^{-1}$ argon flow in the surrounding atmosphere. We identified T$_g$ as the temperature of the endothermic peak in the DSC curve.

### Unconfined vesiculation experiments

To determine the development of porosity and permeability associated with vesiculation of a freely expanding sample, we heated obsidian cores (10 mm/12 mm) at 10 $^\circ$C min$^{-1}$ to 1009 $^\circ$C for different isothermal dwell times of 10–600 min in a Carbolite box furnace. The samples were cooled at a rate of 5 $^\circ$C min$^{-1}$, chosen to minimize the relaxation of the bubble textures, whilst being slow enough to avoid high thermal gradients across the cooling sample which could promote cracking.

The volume of each sample was measured prior to ($V_\text {pre}$) and following ($V_\text {post}$) experimentation via helium pycnometry, to provide the total porosity:

3 $$\begin{aligned} \phi = 1 - \frac{V_\text {pre}}{V_\text {post}} \, . \end{aligned}$$

We note that porosity includes both vesicularity and the void space occupied by fractures that may form both during shear in the experiments and also during cooling-induced contraction. We make the simplifying assumption that the porosity and permeability of our samples is dominated by the vesiculation and that the cooled samples are representative of the microstructure present during the experiment. Additionally, we assume that none of the porosity is connected to the exterior of the sample due to the presence of a dense and impermeable glassy rind formed by diffusive gas loss in a volumetrically insignificant margin around the sample which thickens to about 0.2 mm in 3 hours^59,60^. Following determination of porosity, the samples were prepared for permeability measurements (see Section 5.5).

For a subset of experiments, the samples were imaged through a sapphire window in the furnace using a FLIR thermographic camera (sampling rate of 1 frame min$^{-1}$, spatial resolution of 31.9 ± 4.2 pixels mm$^{-2}$) to monitor sample area (as a proxy for vesicularity) through time. From the images we identified the sample edges, and owing to the cylindrical geometry, we employed the solid of revolution method to geometrically convert the silhouettes into three-dimensional volumes. This approach assumes the sample remains vertically axisymmetric throughout the length of the experiment.

### Confined vesiculation experiments

To determine the impact of spatial confinement on vesiculating magma in a conduit-like geometry, samples were confined in open-topped cylindrical crucibles of dense, holocrystalline basalt. The crucibles all have an external diameter of 25 mm, with various inner diameters of 10.5, 12.7, 14.5, and 18.6 mm. By combining different sample radii (5 or 6 mm), $R_0$, and inner tube radii, $R_c$, we achieved a range of variably confined axisymmetric geometries (see Fig. 1A,B for sketches of the experimental setup). We define the radial confinement ratio, C:

4 $$\begin{aligned} C = \frac{R_0}{R_c} \, \end{aligned}$$

to quantify how much the sample is able to expand isotropically before its margins touch and are impeded by the crucible; any further vesiculation forces expansion to occur along the long axis of the conduit, thus causing anisotropy. When *C *= 1, vesiculation is fully anisotropic, and when $C<$1, vesiculation is first isotropic, then anisotropic.

The samples were placed on a $\sim$1 cm thick, disk-shaped base plate of the same basalt. Prior to the vesiculation experiments, base plates and tubes were thermally treated at 1009 $^\circ$C for 8 h to avoid reaction of the basalt during the experiments. We cemented the basaltic tubes onto the base plates prior to the experiments using a thin film of high-temperature cement to prevent rupture of the assembly during sample vesiculation.

Confined vesiculation experiments followed the same temperature path as the isotropic experiments, with dwell times between 10 and 480 min. The volumes of the samples before and after the experiment were measured along with the basalt crucibles using helium pycnometry as above.

### Permeability and connected porosity measurements

After the porosity determination, samples fused to the crucible were cut at the top and bottom to remove the impermeable rind that forms during diffusive outgassing (Fig. 1C). Unconfined samples and samples not fully coupled to the crucible were cut and measured in a rubber jacket. Permeability through the cut samples was measured using a Vinci Technologies gas permeameter with nitrogen gas, inserted into a compressible Viton jacket and radially loaded to 1 MPa confining pressure using a manual valve. A steady gas flow rate through the sample was selected (0.5 to 125 cm$^3$ min$^{-1}$) to maintain a pressure differential across the sample of $\approx$0.5 psi. The sample permeability was calculated using Darcy’s law with a fluid viscosity for nitrogen at ambient temperature (1.741-1.780$\times 10^{-5}$ Pa s), and the cross-sectional surface area of the vesiculated sample. For very low permeability samples, measurements terminated while the pressure gradient was still increasing, but within the machine tolerance for error and so represent maximum values. The permeability of the basalt crucibles is insignificant compared to the sample permeability; the obsidian has a permeability of 5$\times 10^{-20}$ m$^2$^69^.

After permeability measurements, the cut cores were again measured in the helium pycnometer to determine the connected porosity of the central portion of the sample using the computed cylindrical sample and crucible volumes. With the removed rind, the helium is free to flow into the connected pores and this volume can be compared to the calculated exterior volume in which:

5 $$\begin{aligned} \phi _c = \frac{V_{cut}-\pi H_{cut}(D_o^2-D_i^2)/4}{\pi H_{cut}D_i^2/4} \, , \end{aligned}$$

given the height of the cut sample, $H_{cut}$, and the outer ($D_o$) and interior ($D_i$) of the crucible. If the sample was unconfined or uncoupled to the crucible then:

6 $$\begin{aligned} \phi _c = \frac{V_{cut}}{\pi H_{cut}D_{cut}^2/4} \, \end{aligned}$$

relies only on the height and diameter of the cut sample.

### Textural analysis

We selected two confined and four unconfined samples for backscatter electron (BSE) imaging using a Hitachi TM3000 scanning electron microscope (SEM) at the University of Liverpool. The samples were cut along the vertical axis (parallel to the cylindrical conduit for the anisotropic experiments) using a Well 3500 precision diamond saw. Photos were merged using Adobe Photoshop$\circledR$.

### Vesiculation modeling

We employ the vesiculation model of ^10^ which has been calibrated on the Hrafntinnuhryggur obsidian to recover the mass of water in the bubble. We use the measured major oxides and estimates of bubble number density (3$\times 10^{10}$), with a starting bubble radius of 10 $\upmu$m for numerical stability. Due to the extreme sensitivity of low-pressure vesiculation to small amounts of water loss, we further constrain the starting water content to 0.1235, within the uncertainty on the measured water content to be consistent with the thermal infrared imaging results and post-experimental samples.

We then simulate the heating and cooling path from 709 to 1009 $^\circ$C, followed by dwell times at each experimental condition, and then the cooling path down to 709 $^\circ$C. We then compare the final and peak modeled porosities to quantify the volume change expected during cooling, which is not recoverable from the thermal infrared observations. For samples with short dwell times (<2 h), some net growth occurs during cooling and for samples with longer dwell times ($\ge$2 h) a net loss of porosity occurs during cooling. We correct each measured post-experimental sample to the high-temperature vesicularity on the basis of the modeling (Supplementary Fig. S2). This correction brings the post-experimental unconfined sample volumes into better agreement with the vesicularity measured using the thermographic images, suggesting these results are reasonable. We assume a similar porosity loss/gain occurs in the confined experiments, which are then fit with a curve through the set of observations for each confinement ratio. This curve takes the same shape as the modeled porosity, stretched in both amplitude (total maximum porosity) and time (slowed vesiculation) to best-fit the observations using MatLab’s fminsearch function; below the porosity at which contact with the crucible is established, estimated from the thermographic images, the porosity must follow the unconfined trajectory. This results in piecewise curves for the isotropic and anisotropic portions of the vesicularity evolution.

From the definition of vesicularity and conservation of the bubble number ($N_{b,0}$), we can calculate the radius of the bubble as a function of porosity:

7 $$\begin{aligned} R = \left( \frac{\phi }{1-\phi } \frac{1}{N_{b,0} 4/3 \pi }\right) ^{1/3} \, . \end{aligned}$$

We calculate the two-point numerical derivative and solve eq. 2 for the bubble pressure. This makes the simplifying assumption that the bubble is approximately spherical, which is not the case in our experiments, but should be sufficient for an order-of-magnitude type analysis.

We can express the mass of water per bubble as the water input due to diffusion calculated by the forward model ($\frac{dM_{\text {iso}}}{dt}$) and loss due to permeable escape (Darcy flow):

8 $$\begin{aligned} \frac{dM}{dt} = \frac{dM_\text {iso}}{dt} - \frac{kA (P_b-P_\text {atm})}{\mu _{\text {H2O}} L} \frac{\pi A L}{N_b} \, , \end{aligned}$$

given the bulk permeability, *k*, the cross-sectional area, *A*, the water viscosity, $\mu _{\text {H2O}}$, and sample length, *L*. The cross sectional area and sample length are recovered from the sample volume (porosity) assuming a cylindrical shape and crucible inner diameter. Using the water equation of state from ^70^, we can express the mass of water per bubble on the basis of the bubble pressure and radius and solve explicitly for the permeability as a function of time.

### Sources of uncertainty

We observe scatter in our porosity measurements, especially for experiments at high confinement ratios. The uncertainty from analytical precision on the porosity measurements is an absolute value of 0.001-0.005 cm$^3$, which gives a typical relative uncertainty of <0.1% of the measurement value, so differences in the porosity reflect genuine differences in the experimental products. Bulk porosity is measured using changes between pre-experimental and post-experimental volumes, assuming that the rind at the surface of the samples after the experiments isolates the vesicles from the exterior. However, fractures or breaking of the samples at the base plate may in some cases connect vesicles to the exterior which could result in underestimates of the porosity, especially for samples with high interior connectivity. Indeed the connected porosity on some cut samples exceeds the bulk porosity of those samples prior to cutting, which could reflect either connectivity in the bulk porosity measurement or that the central slice used for permeability measurements avoids a low porosity region at the sample ends as a result of inhibited growth or diffusive volatile loss, although we expect that the latter accounts for the greater portion of differences between connected and bulk porosity. Errors in connected porosity may also arise in the calculation of the cylindrical volume which has potential error from sample faces that are not parallel (typical variation in height around the sample is 0.05 mm leading to an uncertainty in volume and porosity of 0.2-1%), and for the unfused samples, variation in radius (of order 1 mm) which introduces an additional error (typically an overestimate of connected porosity) of approximately 1-3%.

We find reasonable agreement in vesicularity measurements via syn-experimental thermal imaging and post-experimental analysis, although minor disagreements suggest that we may be missing continued deformation during the early stages of cooling. For short duration experiments (<30 min dwell) bubble growth could have continued during the early cooling phase as water should still be diffusing into the bubbles for the first $\sim$50 $^\circ$C and significant bubble overpressures could still be present. In longer duration experiments in which the bubbles have completed most of their growth, cooling could induce bubble shrinkage and a reduction in melt volume (via thermal expansivity of the melt) which could modify the bubble textures^71^, and allow for fracturing or transient disruption of the dehydrated rind which could allow for minor outgassing during cooling. Thermal contraction of the melt phase from the experimental temperature of 1009 $^\circ$C to room temperature (20 $^\circ$C) typically results in a volume reduction of approximately 1%. Similarly, the volume of the gas phase will decrease during cooling; numerical simulations using the model of ^10^, which has been calibrated on the same obsidian predict $\approx$3% bubble shrinkage upon cooling. Empirically, we observe an approximately 5-10% difference in final measured porosity and the porosity determined by imaging (Fig. 4). This also helps explain (along with changes in aspect ratio and outgassing) why some samples are coupled to the crucible walls, despite having porosities slightly below the predicted porosity required to touch the crucible walls (Fig. 5). Additionally, differences in the measurement strategy may provide a systematic offset between the imaging and post-experimental estimates of vesicularity; image analysis uses the 90th percentile of detected pixels while post-experimental analysis is a mean of 10 measurements across the sample (usually mostly near the middle and would have a stronger effect on height than radius).

To quantify the uncertainty on the permeability measurements, we conduct 5 repeated measurements of the permeability on 1 sample and 3 repeat measurements on a further 14 samples from which we can estimate an uncertainty on the measurement precision of $\sim$5% (Supplementary Table 2). The majority of our samples fall within the acceptable limits of Darcy flow. 8 of 106 samples for which permeability was measured had a flow rate rapid enough to warrant correction for the Forchheimer effect. The Klinkenberg effect becomes relevant at very low permeabilities (<10$^{-18}$ m$^2$). None of our samples exhibit measured permeabilities this low, and measurements on samples below $\sim$10$^{-15}$ were terminated before equilibrium conditions were met and, as such, permeability for these samples are only given as maxima (Table 1).

**Table 1:** Major element composition from XRF analysis and water concentration from FTIR of obsidian samples.

Oxide	wt %
SiO$_2$	75.84
TiO$_2$	0.24
Al$_2$O$_3$	12.24
Fe$_2$O$_{3,\text {tot}}$	3.58
MnO	0.11
MgO	0.03
CaO	1.67
Na$_2$O	4.67
K$_2$O	2.68
P$_2$O$_5$	0.02
SO$_3$	<0.001
LOI	0.14
total	101.22
H$_2$O	0.12

## Data Availability

Experimental data will be uploaded to Zenodo upon acceptance. A copy available to reviewers is provided here: https://zenodo.org/records/17697957
